# Equipment to prevent, diagnose, and treat hypothermia: a survey of Norwegian pre-hospital services

**DOI:** 10.1186/1757-7241-21-63

**Published:** 2013-08-12

**Authors:** Anders M Karlsen, Øyvind Thomassen, Bjarne H Vikenes, Guttorm Brattebø

**Affiliations:** 1 Department of Anaesthesia & intensive care, Haukeland University hospital, Bergen, N-5021, Norway

## Abstract

**Introduction:**

Hypothermia is associated with increased morbidity and mortality in trauma patients and poses a challenge in pre-hospital treatment. The aim of this study was to identify equipment to prevent, diagnose, and treat hypothermia in Norwegian pre-hospital services.

**Method:**

In the period of April-August 2011, we conducted a survey of 42 respondents representing a total of 543 pre-hospital units, which included all the national ground ambulance services, the fixed wing and helicopter air ambulance service, and the national search and rescue service. The survey explored available insulation materials, active warming devices, and the presence of protocols describing wrapping methods, temperature monitoring, and the use of warm i.v. fluids.

**Results:**

Throughout the services, hospital duvets, cotton blankets and plastic “bubble-wrap” were the most common insulation materials. Active warming devices were to a small degree available in vehicle ambulances (14%) and the fixed wing ambulance service (44%) but were more common in the helicopter services (58-70%). Suitable thermometers for diagnosing hypothermia were lacking in the vehicle ambulance services (12%). Protocols describing how to insulate patients were present for 73% of vehicle ambulances and 70% of Search and Rescue helicopters. The minority of Helicopter Emergency Medical Services (42%) and Fixed Wing (22%) units was reported to have such protocols.

**Conclusion:**

The most common equipment types to treat and prevent hypothermia in Norwegian pre-hospital services are duvets, plastic “bubble wrap”, and cotton blankets. Active external heating devices and suitable thermometers are not available in most vehicle ambulance units.

## Introduction

Accidental hypothermia represents a challenge in the pre-hospital setting during all seasons and is identified as an independent risk factor for increased mortality and morbidity in trauma patients [1-4]. Trauma patients lose temperature at the accident site, during transport, and in the emergency department [5,6]. The definition of hypothermia is usually a core temperature below 35°C, but there is a considerable individual difference at what temperature the clinical effects occur [7].

Data are limited on the incidence of hypothermia in Scandinavian trauma patients. Studies from the United States and Australia show an incidence of hypothermia in trauma patients from 1.6 to 15.7% [1-3,8]. The risk of hypothermia increases with injury severity and has been reported to occur in over 40% of trauma patients presenting signs of hypoperfusion [9].

Knowledge concerning current treatment practices and use of equipment among rescue personnel for treating and preventing hypothermia outside hospitals is limited and warrants further investigation. One retrospective study of military personnel found that despite introduction of new wrapping concepts and active heating equipment, wool blanket was the most used utility [10]. A survey of American mountain rescue teams found that most teams preferred active warming during evacuation, with chemical heat packs as the dominant method [11]. A survey of EMS personnel in one region in Norway found that wool blankets and hot iv fluids were the most preferred interventions among rescue personnel with hypothermic patients [12].

The aim of this study was to identify equipment available for diagnosis, prevention, and treatment of hypothermia in Norwegian pre-hospital services, defined as the ground ambulance service (AS), helicopter emergency medical service (HEMS), fixed wing air ambulance service (FW), and the national search and rescue service (SAR).

## Methods

The AS is operated through 18 health trusts and conducted approximately 600,000 missions in 2010, serving a population of 4.9 million. The number of ground ambulances was estimated to be around 490 units in daily operation during the study period. HEMS consisted of twelve physician-staffed ambulance helicopters operating from eleven bases, FW of nine planes assigned to seven bases, and SAR had ten helicopters assigned to six bases. Other organisations that handle patients outside the hospital (primary care, non-governmental organisations like the Red Cross, alpine rescue teams, and ski patrols) also provide initial treatment but were not included in this study.

Structured telephone interviews were performed in the period of April-August 2011 and were guided by a questionnaire on the following topics: available insulation materials, active warming equipment, protocols on how to insulate patients, hypothermia thermometers and preferred anatomical measuring sites, pre-warmed i.v. fluids, and infusion heating devices (questionnaire available from corresponding author.). A hypothermia thermometer was defined as a thermometer designed to measure temperature below 32°C. Optional equipment not stored in the units and equipment designed for incubators were excluded. The study was approved by the regional research ethics committee.

In the AS, the primary contact was the head of each health trust’s ambulance department who either responded personally or referred to personnel with better knowledge of the current status. The respondents had to have knowledge of organisational structure and guidelines and detailed knowledge of the current equipment setup in their service. All respondents were asked if there was an equal equipment setup for the whole area and to report the number of ambulance units in their service. In cases of reported variation in equipment setup, a questionnaire was to be distributed electronically to all local station managers in the area. Respondents in the three aero-medical services (FW, HEMS, and SAR) consisted of one of the medical crew members on call.

Data were entered into a standardised database and analysed in a spreadsheet (Microsoft Excel 2010, Microsoft Corp, Redmond, WA, US). Ambiguous answers were interpreted in consensus among the authors, and respondent confirmations were sought when needed.

## Results

We achieved a 100% response rate, with a total of 42 respondents (Table 1). All AS representatives answered that equipment was uniform for all units in their area, and no follow-up at a lower level was needed to complete data.

**Table 1:** Respondents

**Services**	**Number of respondents**	**Total number of units represented by respondents**
Ambulance services	18	512
Fixed wing air ambulance	7	9
Helicopter air ambulance	11	12
Rescue service	6	10
**Total**	**42**	**543**

The dominant insulation materials throughout the services were hospital duvets, plastic “bubble-wrap” and cotton blankets. Wool blankets, “space blankets”, and separate head covers were less frequently reported. Insulation equipment such as insulated shelters and similar insulation materials were absent from most services, with the exception of the SAR services. All SAR bases reported having an insulated shelter bag intended for patient use (Fjellduken®, Jerven as. Odda, Norway). 28% of all AS units had other specially designed insulation products (Table 2).

**Table 2:** Equipment to prevent, diagnose and treat hypothermia in Norway

	**Ground ambulance services (AS)**	**Fixed wing (FW) (n = 9)**	**Helicopter emergency medical service(HEMS)**	**National search and rescue service (SAR)**
	**Total 512**	**Total 9**	**Total 12**	**Total 10**
	**n and (%)**	**n and (%)**	**n and (%)**	**n and (%)**
**Insulation material available**				
Space blankets	151 (29)	5 (56)	8 (67)	0
Hospital duvets	468 (91)	9 (100)	11 (92)	10 (100)
Cotton blankets	436 (85)	7 (78)	7 (58)	2 (20)
Plastic “bubble wrap”	428 (84)	0	12 (100)	10 (100)
Wool blankets	154 (30)	9 (100)	2 (17)	9 (90)
Sleeping bags	0	0	0	0
Shelter bags (not insulated)	0	0	0	0
Insulated shelter bags	0	0	2 (17)	10 (100)
Separate head cover	220 (43)	0	7 (58)	10 (100)
Body bags	512 (100)	0	0	10 (100)
Plastic covers	0	1 (11)	0	0
Other^a^	148 (29)	0	0	0
**Active warming equipment**	72 (14)	4 (44)	7 (58)	7 (70)
Chemical heat pads	57 (11)	4 (44)	7 (58)	5 (50)
Water bottles	0	0	0	5 (50)
Electrical heating blankets	0	0	4 (33)	0
Forced air warmer	0	0	2 (17)	0
Warm i.v. fluid protocol	15 (3)	0	0	0
Other	0	0	0	0
**Treatment protocols**	373 (73)	2 (22)	5 (42)	7 (70)
**Hypothermia thermometers**				
Yes	61 (12)	7 (100)	12 (100)	10 (100)
Not sure	123 (24)	0	0	0
For rectal use	61 (100)^b^	7 (100)	12 (100)	8 (80)
For oesophageal use	0	7 (100)	12 (100)	8 (80)
For tympanic use	46 (75)^b^	0	0	2 (20)
For oral use	0 (0)^b^	1 (11)	0	0
For axillary use	0 (0)^b^	3 (33)	0	0
Other^d^	0	4 (44)	0	3 (30)
**Hot i.v. fluids**	468 (91)	1 (11)	11 (92)^c^	10 (100)
**Infusion heaters**	15 (3)	0	2 (17)	2 (20)

^a^Blizzard® rescue blanket, vacuum mattresses.

^b^Number and percentage of units represented in “Yes” category (n = 61).

^c^HEMS fluids are kept warm in storage lockers at the base, not in the helicopter unit.

^d^Bladder, skin.

18 (43%) of the respondents reported having equipment for providing active warming, which was available in most HEMS (58%) and SAR service units (70%), but to a lesser degree in the FW (44%) and AS (14%) units. Heating pads were most frequent, followed by hot water bottles and heating blankets.

Protocols describing how to insulate patients were present for 73% of AS and 70% of SAR units. The minority of HEMS (42%) and FW (22%) units was reported to have such protocols.

All air services reported having hypothermia thermometers, in contrast to the AS in which 12% of the units had them. The reported anatomical measuring sites were rectal, oesophageal, and tympanic.

Hot i.v. fluids were available in all SAR units and the majority of AS (91%) and HEMS services (92%) but in only one of the FW bases (11%). Infusion warmers were available to a small number of the units (0-20%).

## Discussion

The 100% response rate makes this survey a valid overview of the equipment to prevent, diagnose, and treat hypothermia in the Norwegian pre-hospital services.

The insulation material was largely similar within the SAR and FW services, while greater regional differences emerged within the HEMS and AS. Hospital duvets were present in almost all units. The study also showed that “bubble wrap” were common in all services except FW, which has some insulating effect and also acts as a vapour barrier [13,14].

Active warming equipment was unavailable to most AS units (14%) but fairly common in FW (44%), HEMS (58%), and SAR (70%) units. The main heat source in the survey was heat packs. In the Scandinavian countries there are no current consensus regarding which role active warming devices should have in pre-hospital care. In cases of mild to moderate hypothermia in a patient with intact shivering response, the effect of active warming on core temperature have been questioned [15]. The rate of re-warming will largely be the same as if heat were not added because shivering effectively re-establishes the temperature balance. The main benefits of active re-warming are in these cases to reduce energy consumption and increase comfort [15,16]. In patients with impaired shivering response due to severe hypothermia, injury, disease, intubation or use of drugs, insulation alone may not be sufficient to maintain core temperature and these patients may require active warming [17,18].

Head cover intended for patient use was present in the equipment setup of all SAR bases but to a lesser extent in the other services.

A written procedure describing how to insulate patients who either are hypothermic or in danger of becoming hypothermic was reported by the majority of respondents in the AS (73%) and SAR (70%) services. In HEMS and FW, the minority reported having such a protocol (42%, 22% respectively). The appropriateness of the content and personnel compliance was not included in the study aim and was not assessed. The presence of protocols may be viewed as an indicator of whether or not hypothermia prevention and treatment receives attention.

In addition to clinical signs and environmental clues, a reliable measurement of the core temperature is important in assessing the degree of hypothermia. This study shows that the majority of the AS units lack suitable tools for diagnosing hypothermia. Oesophageal and rectal measurements correspond well with pulmonary artery blood temperature and were the measurement sites reported in the aero-medical services [19]. The ambulance service reported rectal and tympanic temperature as measurement locations. Tympanic measurement without isolation of the ear canal may be inaccurate in the pre-hospital environment, especially if the victim has been submerged [20]. ERC Guidelines 2010 recommend the use of oesophageal, rectal, urinary bladder, or tympanic measurement to determine core temperature in hypothermic patients [21]. A survey of Norwegian rescue workers showed that rectal temperature was the most common measuring site in the vehicle ambulance services, consistent with our findings [12].

Depending on ambient temperature, significant temperature loss occurs in the i.v. tube and infusion bag and is a potential factor for iatrogen heat loss [22-24]. Outside the hospital, it is almost impossible to deliver fluid to patients at near body temperature without specially designed equipment. In most cases, giving intravenous fluids with the intention to re-warm the patient will therefore be counterproductive [25,26]. Heated storage cabinets were reported to be available in almost all AS (91%), HEMS (92%), and SAR units (100%). Infusion heaters were reported only in a small number of the services.

### Limitations

This study has several limitations. We achieved a 100% response to this survey and believe that the data are representative of pre-hospital units in Norway. However, structured interviews are known to suffer from bias. Respondents may provide inaccurate information to give a favourable impression of their service or may remember wrong or misunderstand questions. The interviewer may also unconsciously influence the respondent through his or her behaviour. Equipment is constantly changing, and current status may have changed since the study period.

## Conclusion

The most common equipment types used to treat and prevent hypothermia in Norwegian pre-hospital services are duvets, plastic “bubble wrap”, and cotton blankets. Active external heating devices and suitable thermometers were available in only a few of the ground ambulance services. The findings in this survey may reflect a lack of consensus in how to best prevent and treat hypothermia in the pre-hospital setting. Further research on the effects of the various wrapping concepts is needed.

## Competing interests

The authors declared that they have no competing interests.

## Authors’ contributions

All authors have participated in the development of the protocol and the questionnaire. AMK has performed the interviews and provided the first draft of the article. All authors have been active in the revision and approved the final manuscript.
